# Multiplex Quantitative Analysis of 9 Compounds of *Scutellaria baicalensis* Georgi in the Plasma of Respiratory Syncytial Virus-Infected Mice Based on HPLC-MS/MS and Pharmacodynamic Effect Correlation Analysis

**DOI:** 10.3390/molecules28166001

**Published:** 2023-08-10

**Authors:** Yanghai Wang, Mingqian Jia, Yan Gao, Bonian Zhao

**Affiliations:** Institute of Pharmaceutical Research, Shandong University of Traditional Chinese Medicine, Jinan 250355, China; 2020110277@sdutcm.edu.cn (Y.W.); 2020110272@sdutcm.edu.cn (M.J.)

**Keywords:** *Scutellaria baicalensis* Georgi, HPLC-MS/MS, RSV, pharmacodynamic effect, pharmacodynamic material basis

## Abstract

According to traditional Chinese medicine, *Scutellaria baicalensis* Georgi possesses the therapeutic properties of heat-clearing, dampness-drying, diarrhea alleviation, and detoxification, making it a clinically used remedy for respiratory infections. The objective of this study was to investigate the changes in constituent content, pharmacodynamic effects, and material basis of *Scutellaria baicalensis* Georgi in the plasma of mice infected with respiratory syncytial virus (RSV). The results showed that a sensitive and efficient high-performance liquid chromatography with tandem mass spectrometry (HPLC-MS/MS) method was established in this study. Multiple quantitative analyses of Baicalein, Apigenin-7-glucuronide, Baicalin, Oroxylin A 7-*O*-beta-d-glucuronide, Wogonoside, Norwogonin, Wogonin, Chrysin, and Oroxylin A in mouse plasma revealed a bimodal absorption phenomenon within the time frame of 0.167 h to 6 h post-administration, with the exception of chrysin. Following 6 h of administration, the concentrations of 9 components continued to decrease until they became undetectable. In comparison to the model group, all administered groups exhibited significant reductions in lung index and viral load, with their lung index repair rate and viral suppression rate aligning with the blood concentration-time curve. Finally, through the application of the gray correlation analysis method, we identified Baicalein, Baicalin, Oroxylin A 7-*O*-beta-d-glucuronide, Wogonoside, Norwogonin, and Wogonin as potential pharmacodynamic material bases of *Scutellaria baicalensis* Georgi against RSV infection.

## 1. Introduction

Respiratory syncytial virus (RSV) belongs to the Paramyxoviridae and Pneumovirus families and is a negative-sense, single-stranded RNA virus [1]. It is a prevalent pathogen responsible for respiratory tract infections [2]. RSV infection damages ciliated epithelial cells in the respiratory tract, triggering an exaggerated immune response that leads to injury in the bronchioles, small airways, and alveoli, ultimately resulting in pneumonia. In severe cases, pneumonia can be life-threatening, particularly for vulnerable populations such as infants, elderly individuals, and immunocompromised patients. Hence, RSV infection poses a significant threat to these groups [3,4,5]. Currently, the Food and Drug Administration (FDA) has approved ribavirin [6] and palivizumab [7] for RSV infection treatment. Although these drugs effectively inhibit RSV and alleviate severe symptoms, their use is subject to controversy due to certain limitations [8]. Therefore, there is a critical need to discover safe, effective, and affordable drugs for RSV treatment. Traditional Chinese medicine, with its extensive history and experience in preventing and treating viral infectious diseases, has demonstrated unique advantages in both in vivo and in vitro treatment and holds promising prospects for broad applications. Notably, traditional Chinese medicine has played a significant role in the prevention and treatment of COVID-19. Compound preparations such as Shuanghuanglian preparations, Jinxin oral liquid, Lanqin oral liquid, and Scutellaria Decoction de have shown potential therapeutic effects against viral infections, highlighting the potential of traditional Chinese medicine in combating RSV infection and other viral diseases. Further exploration and research in this area are warranted to develop novel therapeutic interventions that can effectively address the challenges associated with RSV infection.

*Scutellaria baicalensis* Georgi is a perennial herb belonging to the genus Scutellaria in the family Labiatae. It is also known as Camellia sinensis and Tujincha root, with its dried root being used as the medicinal part. The first recorded mention of *Scutellaria baicalensis* Georgi was in the “Shennong Ben Cao Jing”. To date, the majority of research on the anti-RSV infection properties of *Scutellaria baicalensis* Georgi has been pharmacological in nature. *Scutellaria baicalensis* Georgi has the effects of clearing heat and drying dampness, purging fire, and detoxifying, and its clinical prescriptions are commonly used in the treatment of respiratory system diseases, particularly with notable efficacy in combating respiratory tract infections [9]. Li et al. [10] used the 3-(4,5-Dimethylthiazol-2-yl)-2,5-diphenyltetrazolium bromide (MTT) method and the cytopathic degree method to examine the half maximal inhibitory concentration (IC50) of eight batches of aqueous extract samples of *Scutellaria baicalensis* Georgi from different origins and their corresponding mock samples against human laryngeal cancer cells Hep-2 and RSV. They calculated the treatment indexes (TI) and found that the aqueous extracts of *Scutellaria baicalensis* Georgi from different origins had inhibitory effects on RSV. Similarly, Wang et al. [11] used cellular in vitro antiviral assays to derive the half-toxic concentration (TC50) and the concentration for 50% of maximal effect (EC50) by the cytopathic effect (CPE) method combined with the MTT method. They used the therapeutic index as an evaluation index and found that *Scutellaria baicalensis* Georgi extracts had antiviral activity against RSV. Du et al. [12] showed that *Scutellaria baicalensis* Georgi *is* extracts had antiviral activity against RSV by establishing a lipid metabolomics study method in RSV-infected mice. They examined the changes in lipid metabolite groups in plasma and lung tissue samples before and after *Scutellaria baicalensis* Georgi’s intervention. The results showed that RSV infection may be closely related to glycerophosphatidylcholine, sphingomyelin, and triglyceride metabolism. *Scutellaria baicalensis* Georgi’s aqueous extract could play an antiviral role by regulating the disturbed lipid metabolism after intervention. Furthermore, Gao et al. [13] screened the antiviral efficacy of *Scutellaria baicalensis* Georgi using the CPE method and found that it had a significant effect on respiratory syncytial virus. In summary, *Scutellaria baicalensis* Georgi exerts its antiviral effect against RSV infection mainly through two pathways: blocking viral replication and inhibiting biosynthesis.

Pharmacological studies have demonstrated the effectiveness of *Scutellaria baicalensis* Georgi against RSV infection. However, the therapeutic material basis remains unclear. In order to clarify the effective components of *Scutellaria baicalensis* Georgi against RSV infection and its synergistic effect process, it is necessary to clarify the dynamic change process of drug components in vivo. Currently, there is no report on the process of changes in the concentration of components and the pharmacodynamic effects of *Scutellaria baicalensis* Georgi in pathological models of RSV infection. Previously, we employed a combined high-performance liquid chromatography-electrostatic field orbitrap high-resolution mass spectrometry (HPLC-Orbitrap Exploris 120 MS) technique to precharacterize the constituents in the plasma of mice. A total of 9 constituents were identified, including Baicalein, Apigenin-7-glucuronide, Baicalin, Oroxylin A 7-*O*-beta-d-glucuronide, Wogonoside, Norwogonin, Wogonin, Chrysin, and Oroxylin A (Supplementary Table S1, Figure S1). Subsequently, we performed multiple quantitative analyses using the HPLC-MS/MS method. Additionally, we investigated the pharmacodynamic effects of *Scutellaria baicalensis* Georgi on the lung index and viral load in the lung tissues of mice during anti-RSV infection treatment. Finally, employing GRA, we examined the correlation between the dynamic change process of the constituents and the pharmacodynamic effects. This provides a persuasive material basis for elucidating the efficacy of *Scutellaria baicalensis* Georgi, as well as laying the foundation for future studies on its mechanism of action against RSV and drug development.

## 2. Results

### 2.1. Method Validation

#### 2.1.1. Specificity

**Figure 1:**
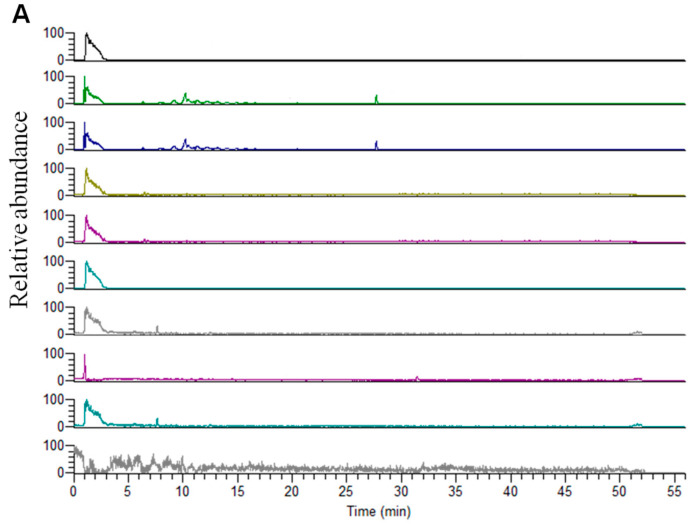

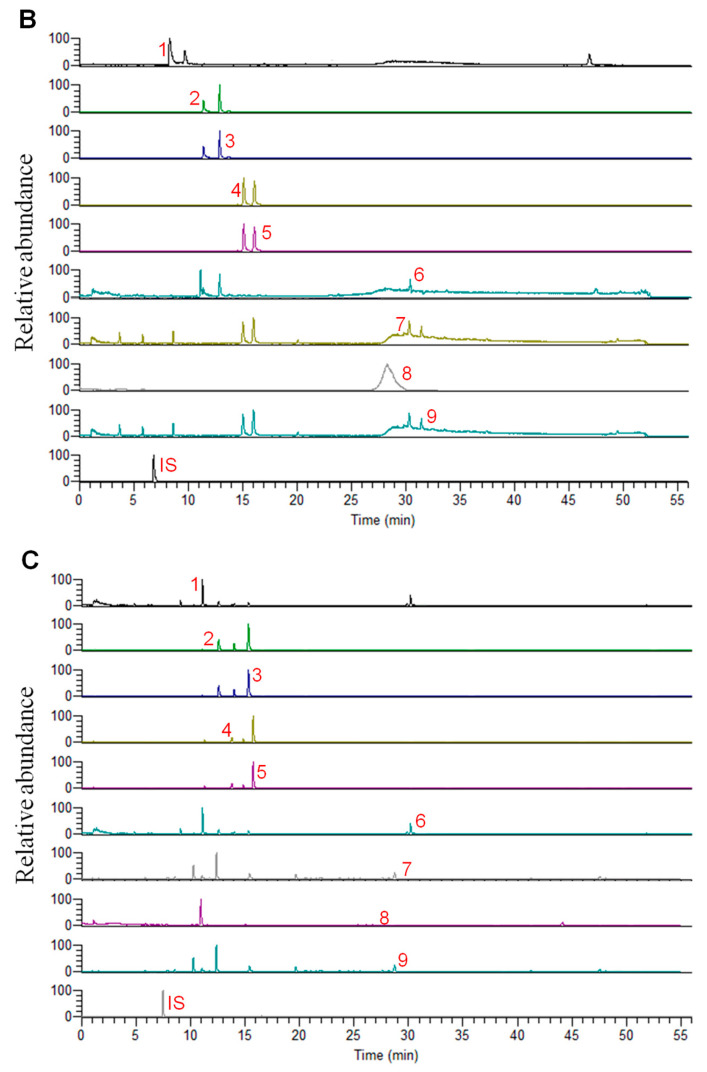
Representative chromatograms of blank plasma samples (**A**), blank plasma spiked with analytes (**B**), and plasma samples collected at 6.0 h after administration of *Scutellaria baicalensis* Georgi (**C**). (1: Baicalein, 2: Apigenin-7-glucuronide, 3: Baicalin, 4: Oroxylin A 7-*O*-beta-d-glucuronide, 5: Wogonoside, 6: Norwogonin, 7: Wogonin, 8: Chrysin, 9: Oroxylin A, IS: Tinidazole).

The chromatograms obtained from mouse blank plasma, blank plasma samples spiked with the analytes, and mouse plasma samples collected 6 h after administration of *Scutellaria baicalensis* Georgi were compared (Figure 1). The comparison demonstrated that there were no significant impurities or endogenous substances that interfered with the plasma, thereby confirming the method’s specificity.

#### 2.1.2. Linearity and Lower Limit of Quantification (LLOQ)

The regression equation, correlation coefficient (R^2^), linear range, and lower limit of quantitation (LLOQ) of 9 compounds in mouse plasma are presented in Table 1. Regression analysis demonstrated a strong linear relationship between the 9 components within the corresponding concentration range (R^2^ ≥ 0.9951). The LLOQ of each analyte was calculated based on a signal-to-noise ratio (S/N) greater than 10. Notably, the LLOQ range for each component was determined to be 1.25 to 12.50 ng/mL, indicating the high sensitivity of the method.

#### 2.1.3. Accuracy and Precision

Table 2 presents a summary of the inter-day and intra-day accuracy and precision of 9 compounds at low, medium, and high concentrations. Precision for all analytes ranged from 1.62% to 9.46%, while accuracy ranged from −13.42% to 12.68%. All values fell within the acceptable range for bioanalysis, with relative error (RE) < ±15% and relative standard deviation (RSD) < 15%. These results demonstrate that the method used was effective in quantifying the 9 analytes with high accuracy and precision.

#### 2.1.4. Extraction Recovery and Matrix Effect

Table 3 presented the extraction recovery and matrix effects of 9 compounds across three concentration levels, namely low, medium, and high. The findings demonstrate that the extraction recoveries of the 9 compounds ranged from 82.52% to 102.69%, while the matrix effects of all compounds varied from 89.61% to 105.52%. 

#### 2.1.5. Stability

As shown in Table 4, the 9 compounds had good stability in mouse plasma under different temperature and time conditions. The RSD% ranges of the 9 compounds were 3.30–11.18%, 2.59–9.92%, and 2.34–9.96% for short-term stability (12 h at 4 °C), freeze-thaw cycling stability (3 times repeated freeze-thaw at −80 °C), and long-term stability (freezed at −20 °C for 20 days), respectively.

#### 2.1.6. Pharmacokinetics Experiment

**Figure 2:**
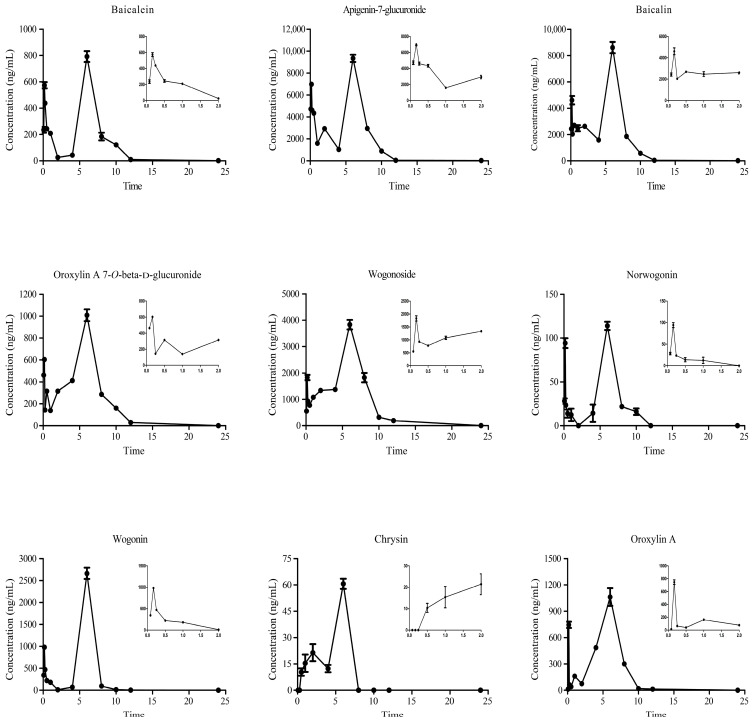
The plasma concentration-time curves of the 9 target components of *Scutellaria baicalensis* Georgi.

In the present study, a multiplexed quantitative analysis of 9 compounds in mouse plasma was carried out by HPLC-MS/MS. The drug concentration-time curves and data for each analyte are presented in Figure 2 and Table S2, respectively. The results indicate that the concentration-time curve of the 8 compounds (except Chrysin) in the blood exhibited a biphasic absorption pattern within the 0.167~6 h period following the administration of *Scutellaria baicalensis* Georgi. The drug concentration began to decline significantly after 6 h and remained at a low level after 8 h. After 12 h, the drug content was nearly undetectable.

### 2.2. Pharmacodynamic Experiment

**Figure 3:**
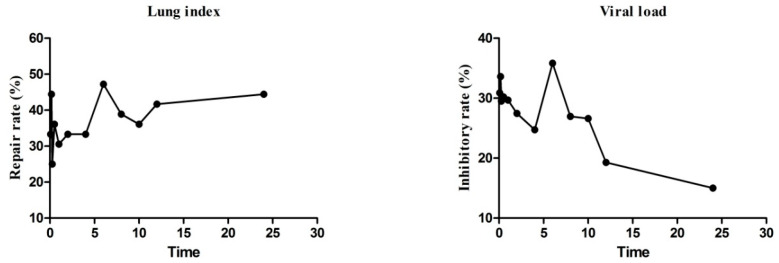
The efficacy index change curve of *Scutellaria baicalensis* Georgi.

The efficacy of *Scutellaria baicalensis* Georgi administration in treating RSV-infected mice was evaluated by examining the drug’s effectiveness over time, as illustrated in Figure 3 and Table 5. The findings indicate that the lung index repair rate and viral load inhibition rate both peaked at 0.167 h and 6 h after *Scutellaria baicalensis* Georgi administration, presumably related to the secondary absorption of active ingredients by *Scutellaria baicalensis* Georgi after entering the hepatic and intestinal circulation via biliary excretion. However, after 10 h, the viral inhibition rate of *Scutellaria baicalensis* Georgi gradually decreased with time. These results demonstrate that *Scutellaria baicalensis* Georgi effectively inhibits inflammation and reduces the viral load in the lungs of RSV-infected mice. 

### 2.3. Related Analysis

#### 2.3.1. Combined Weight Score of Efficacy Indicators

Information entropy is a probabilistic expression of discrete values. According to its definition, the entropy value can determine the degree of discreteness of an indicator. A smaller information entropy value indicates a greater degree of discreteness, suggesting that the indicator has a stronger influence on the comprehensive evaluation [14,15]. Therefore, the entropy weighting method can be used to objectively assign the discriminative coefficients of each index and calculate them through weights in order to objectively and accurately reflect various types of information. In this study, the efficacy index (E%) of lung index and viral load were subjected to the entropy method weighting composite score, as shown in Table 6.

#### 2.3.2. Grey Relation Analysis

The Grey Relation Analysis (GRA) is a multi-factor statistical analysis method used to measure the similarity or dissimilarity of development trends among multiple factors. The greater the correlation degree, the stronger the correlation between the comparative factors and the reference factors [16]. A correlation degree ≥ 0.9 indicates a significant effect of the subsequence on the parent sequence, while a correlation degree between 0.8 and 0.9 indicates a relatively significant effect. In this study, the comprehensive potency index S was chosen as the reference value (parent sequence), and the values of the 9 compounds of *Scutellaria baicalensis* Georgi at different times after administration were used as the characteristic sequences. The gray correlation formula was applied to calculate the correlations, and the results are presented in Table 7. The results showed that each compound of *Scutellaria baicalensis* Georgi had a significant impact on S.

## 3. Discussion

The pharmacological effects of Traditional Chinese Medicine (TCM) may arise mainly from its key components rather than all the chemical components contained in TCM, due to the significant differences in biological activity, chemical properties, and content of the various components [17]. Effective ingredients of TCM, administered orally, must be transported to the target by blood as a carrier, making absorption into the blood crucial. The application of Liquid Mass Spectrometry (LMS) in the qualitative and quantitative analysis of TCM can help identify chemical components in drug-containing serum comprehensively and accurately, providing strong data support for the basic research of TCM efficacy substances. In this study, we used HPLC-MS/MS combined technology to quickly, efficiently, and sensitively quantify 9 compounds in *Scutellaria baicalensis* Georgi anti-RSV-infected mice and investigated their methodology. The results of the methodological investigation showed that the linear relationship was good (R^2^ > 0.990), and intra-day/intra-day precision, intra-day/intra-day accuracy, repeatability, stability, extraction recovery, and matrix effect all met the quantitative requirements of biological samples stipulated by FDA.

Using HPLC-MS/MS technology, we conducted the content determination and pharmacokinetic analysis of 9 compounds at 12 different time points. The results of the content determination showed that the RSV-infected mice had the most drug-containing components in the blood and the highest content of each component at 6 h after the treatment of *Scutellaria baicalensis* Georgi. The blood concentration-time profiles showed that Baicalein, Apigenin-7-glucuronide, Baicalin, Oroxylin A 7-*O*-beta-d-glucuronide, Wogonoside, Norwogonin, Wogonin, and Oroxylin A reached their highest concentrations at 0.167 h and 6 h, showing a double peak phenomenon consistent with previous literature findings. This may be due to the excretion of *Scutellaria baicalensis* Georgi’s original components through bile, which then enter the hepatoenteric circulation [18,19,20,21]. After the second absorption, the concentration of the 9 compounds showed a significant decrease, remaining at low levels after 8 h and becoming almost undetectable after 12 h, consistent with the pharmacokinetic results of some components in rats after oral administration of the Scutellaria-coptis herb couple and Shuanghuanglian oral liquid [22,23].

The pharmacodynamic test results demonstrated that *Scutellaria baicalensis* Georgi administration significantly improved both lung index repair rate and lung tissue virus inhibition rate, which were markedly different from the model group. This suggests that inflammation and virus infection in the lungs of mice were suppressed. The drug typically enters the human body within 0–6 h during the absorption phase, and the blood concentration rises continuously. At 0.083–2 h of administering *Scutellaria baicalensis* Georgi, the drug concentration reaches its peak, and the pulmonary index repair rate and viral inhibition rate reach their maximum at 0.167 h. However, with the passage of time, the pulmonary index repair rate and viral inhibition rate gradually decrease. Upon observing the 3–6 h time period after administration, the pulmonary index repair rate and viral inhibition rate again appear to increase. This phenomenon may be attributed to the hepatic-intestinal circulation. The viral inhibition rate decreases after 10 h, possibly due to the significant decrease in blood concentration of *Scutellaria baicalensis* Georgi in the body. However, the lung index repair rate increases slightly, likely due to the anti-inflammatory effect of the mice themselves, which helps repair lung tissue. The efficacy-time curve suggests a high correlation between changes in the anti-RSV efficacy index of *Scutellaria baicalensis* Georgi and changes in blood concentrations of its compounds. As *Scutellaria baicalensis* Georgi has many active compounds, further correlation analysis using in vivo data are necessary to determine the pharmacodynamic basis of *Scutellaria baicalensis* Georgi’s anti-RSV infection.

The pharmacodynamic material basis of traditional Chinese medicine refers to the general term for all active ingredients in traditional Chinese medicine that exert clinical efficacy, and its research is the key to modern research in traditional Chinese medicine. There are many research methods for determining the material basis of the efficacy of traditional Chinese medicine. Among them, serum pharmacochemistry can be based on the overall and comprehensive analysis of serum components, select appropriate mathematical analysis methods to analyze the concentration changes of serum components and the changes in efficacy, and focus on the in vivo action process of active components so as to more deeply and accurately clarify the material basis of the efficacy of traditional Chinese medicine [24].

GRA is a suitable analysis method for samples with a non-normal distribution. GRA is suitable for correlation analysis of various factors in small samples and can determine the degree of correlation between components and drug efficacy. It also reflects the contribution of each compound to the drug’s effect. Therefore, the GRA analysis method was used for the data analysis of *Scutellaria baicalensis* Georgi anti-RSV infection samples that did not conform to normal distribution to clarify the pharmacodynamic material basis of *Scutellaria baicalensis* Georgi anti-RSV. GRA analysis results showed that the correlation degree between the 6 components (Baicalein, Baicalin, Oroxylin A 7-*O*-beta-d-glucuronide, Wogonoside, Norwogonin, and Wogonin) and S was greater than 0.9. Finally, the selection basis was “the gray correlation degree is greater than 0.9”. This confirmed that Baicalein, Baicalin, Oroxylin A 7-*O*-beta-d-glucuronide, Wogonoside, Norwogonin, and Wogonin are the pharmacoactive substances in *Scutellaria baicalensis* Georgi against RSV.

Flavonoids and their glycosides are the main active components of *Scutellaria baicalensis* Georgi anti-viruses (RSV, Dengue virus, SARS-CoV-2, etc.), including Baicalein, Baicalin, Oroxylin A 7-*O*-beta-d-glucuronide, Wogonin, etc. The main clinical manifestation of RSV infection is an inflammatory response. It has been reported that Baicalein can exert anti-inflammatory effects by activating estrogen receptors and inhibiting the activation of NF-κB signaling pathways [25]. In addition, in the RSV virus infection model, Meng et al. [26] found that the liver protective effect of Baicalein was mainly related to the metabolic pathways of aspartic acid, glycine, and phosphoinositide based on the metabolomics technology of gas chromatography-mass spectrometry (GC-MS). Studies have shown that in the RSV virus infection model, Baicalin exerts a therapeutic effect by blocking RSV virus attachment, inhibiting viral replication, reducing the expression of viral RSV-G and F proteins, reducing the infiltration of macrophages and T lymphocytes into the lungs, and inhibiting the expression of pro-inflammatory factors [27]. In addition, Li et al. [28] summarized the antiviral properties of Baicalin and found that in the RSV virus infection model, the antiviral effect of Baicalin may be related to the following pathways and proteins, including activation of the JAK/STAT signaling pathway (activation of JAK-1, TYK-2, promotion of STAT1/2 phosphorylation), up-regulation of IFNs, and SOCS1/3 protein expression levels. Ma et al. [29] found that *Scutellaria baicalensis* Georgi extract had good antiviral activity against the RSV virus and that Wogonin was one of the active components against RSV (IC_50_ = 7.4 μg/mL). Further studies have shown that Wogonin can induce Nrf2 activation, enhance the expression of antioxidant enzymes, and inhibit the NF-κB signaling pathway, thereby improving the inflammatory response [30]. In addition, in the RSV virus infection model, the clinical manifestations of RSV in Nrf2-deficient mice were significantly increased, mainly related to the Nrf-2-ARE-mediated antioxidant pathway. Nrf-2-ARE can play an anti-RSV infection role by up-regulating antioxidants, enhancing virus clearance, and regulating inflammatory mediators [31]. Traditional Chinese medicine has the characteristics of a multi-component, multi-target, multi-pathway, and synergistic effect. Therefore, the active components of v inhibit the replication of the virus, inhibit the expression of inflammatory response-related signaling pathways and proteins, and reduce the release of inflammatory factors through synergistic effects, thereby exerting anti-RSV virus effects.

## 4. Materials and Methods

### 4.1. Materials and Chemicals

The *Scutellaria baicalensis* Georgi was obtained from the *Scutellaria baicalensis* Georgi plantation base in Jinan, Shandong province (Jinan, China), while the ribavirin granules (New Bolin), Lianhua Qingwen granules, and Baicalein were purchased from Sichuan Baili Pharmaceutical Co., Ltd. (Chengdu, China), Beijing Yiling Pharmaceutical Co., Ltd. (Beijing, China), and Chengdu Manstead Biotechnology Co., Ltd. (Chengdu, China), respectively. Additionally, Apigenin-7-glucuronide and Wogonoside were obtained from Shanghai Yuanye Biotechnology Co., Ltd. (Shanghai, China), whereas Baicalin was purchased from the China Institute for the Control of Pharmaceutical and Biological Products. Similarly, Oroxylin A 7-*O*-beta-d-glucuronide, Norwogonin, Wogonin, and Chrysin were purchased from Chengdu Pusi Biotechnology Co., Ltd. (Chengdu, China). The thousand-layer paper element A was obtained from Shanghai Shidande Standard Technology Service Co., Ltd. (Shanghai, China). Furthermore, the respiratory syncytial virus (RSV) was provided by the Shandong Academy of Medical Sciences (Jinan, China), and the isoflurane anesthetic was purchased from Shanghai Yuyan Scientific Instrument Co., Ltd. (Shanghai, China). Tinidazole was purchased from Shanghai Yuanye Biotechnology Co., Ltd. The SPARKeasy tissue/cell RNA rapid extraction kit was purchased from Shandong Sikejie Biotechnology Co., Ltd. (Rizhao, China). The reverse transcription kit and Real Time RT-PCR kit were purchased from TaKaRa Baori Medical Biotechnology (Beijing, China) Co., Ltd. PCR primers were purchased from Shenggong Biotechnology (Shanghai, China) Co., Ltd. 

### 4.2. Instrumentation and Conditions

The study employed a Vanquish high-performance liquid chromatograph for chromatographic separation, with a HALO C18 column (2.1 × 100 mm, 2.7 μm) as the chromatographic column. The mobile phase was composed of 0.1% formic acid aqueous solution (A) and 0.1% formic acid acetonitrile solution (B), using a gradient elution of: 0–10 min, 95–75% A; 10–25 min, 75–60% A; 25–27 min, 60–45% A; 27–47 min, 45–30% A; 47–51 min, 30–0% A; 51–53 min, 0–95% A; 53–56 min, 95% A. The column temperature was maintained at 30 °C with a flow rate of 0.3 mL/min and an injection volume of 3.0 μL. 

**Figure 4:**
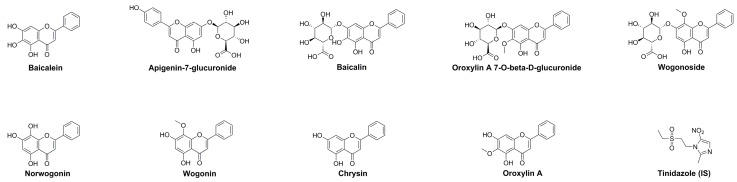
Chemical structures of the 9 compounds and internal standards (IS).

Mass spectrometry was performed using a quadrupole-electrostatic field orbitrap desktop mass spectrometer equipped with an electrospray ion source (ESI). The ESI parameters were set as follows: the pyrolysis voltage was 3.0 kV, the auxiliary gas flow rate was 10 mL/min, the ion transport tube temperature was 350 °C, the auxiliary gas temperature was 350 °C, and the sheath gas flow rate was 45 L/min. Quantitative analysis was performed using the selective ion detection (SIM) mode (Table S1). The chemical structures of the 9 compounds are shown in Figure 4.

### 4.3. Preparation of Sample Solutions

First, 2.836 g of *Scutellaria baicalensis* Georgi powder were weighed precisely and placed in a round-bottom flask. Then, boiling water was added to the flask at a material-liquid ratio of 1:80, and the mixture was heated and extracted at reflux for 40 min, being shaken every 10 min. After extraction, the flask was removed and cooled to room temperature before being weighed again. The lost weight was then made up with distilled water, and the resulting mixture was filtered and centrifuged at a speed of 2500 revolutions per minute for 15 min. The supernatant was then concentrated in an evaporating dish and transferred to a 10 mL volumetric flask, which was fixed to the scale and stored in a centrifuge tube for freezing. The final concentration was 283.6 milligrams per milliliter.

### 4.4. Animal Grouping and Plasma Sample Preparation

Male BALB/c mice, aged 2 weeks and weighing 10–12 g, were chosen for this study. They were acclimatized for 2 days and then randomly assigned to one of five groups: blank (C), model (M), positive control ribavirin (Y_1_), positive control lianhua qingwen granules (Y_2_), and *Scutellaria baicalensis* Georgi administration (H1-H12). Each group consisted of six mice, and their weights were recorded. The mice were pre-administered for 2 days, and on the third day, RSV virus (TCID50 = 10^−6^) was inoculated through intranasal infection at a volume of 35 μL per mouse. The blank group was inoculated with normal saline of the same volume. After the inoculation of the RSV virus, the blank and model groups were given distilled water by gavage, while the drug-administered groups were given an aqueous extract of *Scutellaria baicalensis* Georgi (10 µL/g) once a day for 3 days. The mice were observed daily for their mental status, hair color, diet, and body weight. Blood was extracted from the eyes at 0.083, 0.167, 0.25, 0.5, 1, 2, 4, 6, 8, 10, 12, and 24 h after the last administration, and the whole blood was placed in a centrifuge tube containing sodium heparin. It was then centrifuged at 3000 r/min for 10 min, and the upper layer of plasma was stored at −80℃. Lung tissues were weighed, the lung index was calculated, and the lung tissues were stored in the refrigerator at −80 °C for freezing.

The plasma sample (200 μL) was processed in a 5 mL tube. Subsequently, 10 μL of 5% formic acid and 20 μL of 2 mol/L hydrochloric acid were added, followed by vortexing for 1 min with 10 μL of internal standard solution (100 ng/mL tinidazole). The extraction was completed by adding 2000 μL of acetonitrile solvent and vortexing for 2 min. The mixture was then centrifuged at 12,000 r/min at 4 °C for 10 min, and the supernatant solution was collected in a new centrifuge tube. The supernatant solution was dried by nitrogen at room temperature before being redissolved in 50 μL of 70% acetonitrile solution containing 0.1% formic acid. After vortexing for 1 min, the solution was centrifuged at 12,000 r/min at 4 °C for 10 min, and the resulting supernatant was injected for analysis. The injection volume was 3 μL. 

### 4.5. Stock Standards and Quality Control (QC) Samples

Each reference substance was precisely weighed and dissolved in 70% methanol to obtain the stock solution. The resulting stock solution was then gradually diluted with 70% methanol to prepare a series of required reference solutions. Table 8 displays the gradient of each reference solution as well as the concentration of the quality control samples (low, medium, and high).

Ribavirin (0.07566 g) was precisely weighed and placed in a 10 mL volumetric flask. It was then diluted with distilled water to the desired volume, sonicated for 30 min, and cryopreserved.

For the Lianhua Qingwen granules, a precise weighing of 3.0266 g was conducted and placed in a 10 mL volumetric flask. Distilled water was added to reach the desired volume, followed by 30 min of ultrasonic treatment. The mixture was then cryopreserved.

### 4.6. Method Validation

The specificity, linearity, lower limit of quantification (LLOQ), accuracy, precision, extraction recovery, matrix effect, and stability of the HPLC-MS/MS method were verified according to the Food and Drug Administration Guidance for Bioanalytical Method Validation (Food and Drug Administration, 2018) [32].

### 4.7. Pharmacokinetics Experiment

The drug-containing plasma at different time points was subjected to sample treatment. The HPLC-MS/MS analysis method was used to quantitatively detect the 9 compounds in the ion detection (SIM) mode. According to the relative peak area value = index peak area/internal standard peak area, the corresponding concentration value was calculated by substituting the standard curve of each component.

### 4.8. Pharmacodynamic Experiment

Based on the recorded body weight and lung tissue weight of mice in the blank, model, positive control, and administration groups, the lung index and lung index repair rate were calculated for each group. A graph depicting the change in lung index repair rate over time was plotted, with time as the abscissa and lung index repair rate data as the ordinate.

The F protein is a RSV-specific protein, so it can be used as a PCR gene indicator to indicate the viral load of lung tissue. The lung tissues of mice from each group were homogenized with lysis buffer to extract total RNA. After quantification, the RNA was reverse transcribed into cDNA, and the SYBR Green Master Mix reagent was used as the reaction system. Real-time polymerase chain reaction (PCR) was performed using the Bio-Rad CFX Connect real-time PCR instrument, with glyceraldehyde-3-phosphate dehydrogenase (GAPDH) serving as the internal reference. The viral load inhibition rate was calculated using the 2^−ΔΔCt^ formula, and a graph depicting the change in lung tissue viral load over time was plotted, with time as the abscissa and lung tissue viral load as the ordinate.

The primer sequences used were as follows: RSV-F: upstream: GCAACCAACAATCGAGCCAG, downstream: GGCGATTGCAGATCCAACACC; GAPDH: upstream: GGGTCCCAGCTTAGGTTCATC, downstream: CCAATACGGCCAAATCCGTTC.

### 4.9. Correlation Analysis

#### 4.9.1. Comprehensive Weight Score of Lung Index and Viral Load for Pharmacodynamic Index

The entropy method was utilized to calculate the composite score for the efficacy index E% of the lung index and viral load. Initially, the dimensionless data standardization of the original data were carried out so that the dimension of each index was unified. Subsequently, the characteristic weight, information entropy, and weight of each indicator were computed. Finally, the comprehensive score S for each sample evaluation index was obtained.

#### 4.9.2. Grey Correlation Analysis

The original data for each group was dimensionless, using the averaging method. The comprehensive efficacy index S was set as the parent sequence reference value. The content values of 9 compounds of *Scutellaria baicalensis* Georgi at different time points after administration were designated as the characteristic sequence. These values were then input into the gray correlation calculation formula to determine the degree of correlation.

### 4.10. Data Analysis

GraphPad Prism 8.0 software was used to plot blood concentration-time profiles, graphs of changes in pharmacodynamic indicators, data matrices, and data analysis. 

Other parameters, including lung index, lung index repair rate, and viral load inhibition rate, were calculated using the following equations: lung index (%) = lung mass/mouse body mass × 100%; lung index repair rate (%) = (lung index of model group − lung index of administered group)/(lung index of model group − lung index of blank group) × 100% conversion; viral load inhibition rate (%) = (viral load of model group − viral load of administered group viral load)/viral load of the model group × 100%.

## 5. Conclusions

This study utilized HPLC-MS/MS to quantify the 9 compounds of *Scutellaria baicalensis* Georgi in the plasma of RSV-infected mice as well as to study the changes in the content of each component in mice, and it was found that all 9 compounds showed a double peak phenomenon. The pharmacodynamic study demonstrated that the administered group significantly reduced the lung index and viral load at different time points. No significant difference was observed between the groups, indicating that *Scutellaria baicalensis* Georgi could inhibit the inflammatory response in the lungs of mice. Correlation analysis suggested that Baicalein, Baicalin, Oroxylin A 7-*O*-beta-d-glucuronide, Wogonoside, Norwogonin, and Wogonin may be the main active components of *Scutellaria baicalensis* Georgi against RSV infection. Pathological conditions significantly affect the pharmacokinetic processes of drugs in the organism, as evidenced by previous studies [33,34,35]. Therefore, the results of this study provide a theoretical basis for the mechanistic study of *Scutellaria baicalensis* Georgi and the development of clinical anti-RSV drugs.

## Figures and Tables

**Table 1:** Regression equation, R2, Linear range and LLOQ of each compound.

Compounds	Regression Equation	R^2^	Linear Range (ng/mL)	LLOQ (ng/mL)
Baicalein	y = 0.00004x + 0.0031	0.9989	1.25–50,000.00	1.25
Apigenin-7-glucuronide	y = 0.0015x + 0.7049	0.9980	1.25–50,000.00	1.25
Baicalin	y = 0.0028x + 1.4684	0.9951	1.25–50,000.00	1.25
Oroxylin A 7-*O*-beta-d-glucuronide	y = 0.0036x − 0.0948	1.0000	1.25–50,000.00	1.25
Wogonoside	y = 0.0072x + 0.6043	0.9996	6.25–25,000.00	6.25
Norwogonin	y = 0.0003x + 0.1517	0.9974	12.50–50,000.00	12.50
Wogonin	y = 0.00007x + 0.0385	0.9986	1.25–50,000.00	1.25
Chrysin	y = 0.0002x + 0.0752	0.9990	12.50–50,000.00	12.50
Oroxylin A	y = 0.00007x − 0.0140	0.9981	12.50–50,000.00	12.50

**Table 2:** Inter-day and intra-day precision and accuracy of 9 compounds at low, medium, and high concentrations (*n* = 6).

Compounds	Nominal Concentration (ng/mL)	Intraday		Interday	
		Precision (RSD%)	Accuracy (RE%)	Precision (RSD%)	Accuracy (RE%)
Baicalein	12.5	4.02	3.13	4.28	0.68
	320.0	3.59	6.90	6.43	6.37
	20,000.0	3.22	5.46	2.52	6.24
Apigenin-7-glucuronide	12.5	3.20	−5.04	2.89	−13.42
	320.0	2.84	−1.74	3.57	−0.80
	20,000.0	3.62	8.80	3.71	11.23
Baicalin	12.5	3.86	−8.26	2.23	−8.64
	320.0	3.37	−3.09	2.95	−5.32
	20,000.0	2.62	3.53	3.79	5.71
Oroxylin A 7-*O*-beta-d-glucuronide	12.5	5.18	10.79	5.26	2.05
	320.0	4.22	2.11	3.30	1.56
	20,000.0	2.68	−0.41	2.96	−0.76
Wogonoside	12.5	4.95	−4.34	3.86	−12.82
	160.0	3.16	−1.67	3.44	1.83
	10,000.0	2.93	4.49	1.62	4.44
Norwogonin	25.6	5.35	−7.47	7.16	−4.28
	320.0	8.77	3.45	6.67	7.05
	20,000.0	7.35	3.72	5.94	3.22
Wogonin	12.5	6.50	−1.30	6.75	−8.77
	320.0	5.54	12.68	4.66	9.59
	20,000.0	7.20	9.78	7.29	7.66
Chrysin	25.6	8.13	−4.89	5.16	−4.59
	320.0	6.30	−9.08	5.65	−3.27
	20,000.0	5.25	−3.70	5.52	−2.40
Oroxylin A	25.6	9.46	−1.64	7.40	−4.83
	320.0	6.79	−9.20	8.88	−6.39
	20,000.0	7.93	−5.18	7.11	−6.12

**Table 3:** Extraction recoveries and matrix effects for 9 compounds at high, medium, and low concentrations (*n* = 6).

Compounds	Nominal Concentration (ng/mL)	Recovery		Matrix Effect	
		Mean ± SD (%)	RSD (%)	Mean ± SD (%)	RSD (%)
Baicalein	12.5	82.52 ± 4.39	5.02	91.04 ± 1.98	2.04
	320.0	90.83 ± 6.81	7.50	89.61 ± 3.91	4.32
	20,000.0	94.69 ± 6.73	7.11	95.36 ± 3.73	3.92
Apigenin-7-glucuronide	12.5	96.51 ± 5.78	5.99	94.62 ± 4.82	4.92
	320.0	98.05 ± 4.92	5.02	98.39 ± 2.63	2.68
	20,000.0	101.01 ± 4.89	4.84	100.04 ± 2.15	2.15
Baicalin	12.5	92.59 ± 4.10	4.43	97.95 ± 2.75	2.77
	320.0	102.12 ± 3.33	3.26	101.71 ± 1.07	1.05
	20,000.0	98.11 ± 3.01	3.07	98.14 ± 1.04	1.06
Oroxylin A 7-*O*-beta-d-glucuronide	12.5	92.75 ± 5.41	5.84	93.22 ± 2.28	2.45
	320.0	95.27 ± 2.31	2.43	95.83 ± 7.36	7.42
	20,000.0	97.16 ± 3.02	3.10	97.58 ± 1.48	1.52
Wogonoside	12.5	93.39 ± 3.28	3.51	97.57 ± 2.96	3.04
	160.0	94.91 ± 4.72	4.97	100.73 ± 1.79	1.77
	10,000.0	99.50 ± 3.52	3.54	105.52 ± 1.81	1.76
Norwogonin	25.6	88.75 ± 2.39	8.71	92.98 ± 1.30	1.40
	320.0	84.19 ± 9.57	11.36	90.99 ± 3.50	3.65
	20,000.0	92.48 ± 9.57	10.58	89.95 ± 4.76	5.29
Wogonin	12.5	91.72 ± 5.35	6.38	93.11 ± 3.90	4.19
	320.0	85.07 ± 2.56	2.87	97.12 ± 1.45	1.49
	20,000.0	102.69 ± 6.62	6.44	95.18 ± 1.38	1.45
Chrysin	25.6	82.58 ± 3.57	4.07	93.84 ± 3.26	3.47
	320.0	91.24 ± 7.31	8.01	91.09 ± 0.98	1.08
	20,000.0	95.41 ± 3.87	4.05	96.00 ± 2.15	2.04
Oroxylin A	25.6	88.74 ± 7.23	7.72	92.75 ± 6.21	6.38
	320.0	94.71 ± 5.85	6.18	95.63 ± 2.33	2.44
	20,000.0	101.20 ± 7.48	7.39	95.27 ± 4.29	4.50

**Table 4:** Stability of 9 compounds under different conditions (*n* = 6).

Compounds	Nominal Concentration (ng/mL)	Short-Term		3-Freeze-Thaw Cycles		Long-Term	
		RSD (%)	RE (%)	RSD (%)	RE (%)	RSD (%)	RE (%)
Baicalein	12.5	4.03	0.16	5.88	5.27	6.03	0.35
	320	6.23	8.44	6.03	2.37	7.95	6.19
	20,000	3.71	4.32	4.82	2.97	3.47	4.32
Apigenin-7-glucuronide	12.5	4.62	−3.20	4.84	−2.43	4.70	−3.16
	320	3.45	−2.08	4.11	−0.72	4.75	−1.48
	20,000	5.41	8.60	6.79	8.37	7.27	9.80
Baicalin	12.5	4.34	−9.16	3.42	−7.38	4.86	−8.15
	320	3.73	−5.85	2.59	−2.37	3.23	−5.21
	20,000	3.30	5.73	4.25	7.69	3.42	5.87
Oroxylin A 7-*O*-beta-d-glucuronide	12.5	4.54	7.34	5.41	6.23	5.70	7.73
	320	5.10	1.89	4.31	3.08	4.21	2.21
	20,000	3.99	−0.23	3.28	−0.56	2.34	0.55
Wogonoside	12.5	4.46	−3.08	5.23	−8.61	5.36	−2.30
	160	4.94	−1.39	5.09	0.60	5.25	−2.01
	10,000	3.84	3.40	5.20	1.79	5.43	3.40
Norwogonin	25.6	8.16	−2.17	7.99	−6.11	5.36	−8.96
	320	6.94	0.74	8.69	7.06	9.29	3.74
	20,000	8.72	−1.39	9.92	1.97	9.96	0.11
Wogonin	12.5	11.18	−9.77	8.75	−11.16	7.17	−13.33
	320	7.66	11.75	5.36	9.82	6.21	11.75
	20,000	8.79	8.46	5.84	12.89	7.05	8.46
Chrysin	25.6	7.49	−5.59	8.78	−6.42	7.46	−4.52
	320	5.18	−10.49	8.69	−5.15	4.46	−9.37
	20,000	6.34	−2.74	5.58	−2.47	4.95	−3.86
Oroxylin A	25.6	4.36	−3.30	5.86	−4.26	8.81	−6.71
	320	5.22	−5.14	6.60	−8.41	6.49	−5.78
	20,000	6.84	−4.07	9.34	−5.29	6.57	−7.03

**Table 5:** Results of lung index repair rate in each group of mice (mean ± SD, *n* = 6).

Group	Lung Index (%)	Repair Rate (%)	Viral Load (×10^5^ Copies/Ml)	Inhibitory Rates (%)
C	0.70 ± 0.06		—	—
M	1.06 ± 0.20 ^∆∆^		5.86 ± 0.40	—
Y_1_	0.85 ± 0.08 **	58.33	2.27 ± 0.37 **	61.26
Y_2_	0.86 ± 0.14 **	55.56	3.52 ± 0.71 **	39.93
0.083	0.94 ± 0.05 *	33.33	4.05 ± 0.39 **	30.89
0.167	0.90 ± 0.12 **	44.44	3.89 ± 0.19 **	33.62
0.25	0.97 ± 0.10	25.00	4.13 ± 0.51 *	29.52
0.5	0.93 ± 0.10 *	36.11	4.09 ± 0.59 *	30.20
1	0.95 ± 0.08	30.56	4.12 ± 0.33 *	29.69
2	0.94 ± 0.09 *	33.33	4.25 ± 0.36 *	27.47
4	0.94 ± 0.16 *	33.33	4.41 ± 0.17 *	24.74
6	0.89 ± 0.10 **	47.22	3.76 ± 0.29 **	35.84
8	0.92 ± 0.06 **	38.89	4.28 ± 0.41 *	26.96
10	0.93 ± 0.03 *	36.11	4.30 ± 0.54 *	26.62
12	0.91 ± 0.07 **	41.67	4.73 ± 0.09	19.28
24	0.90 ± 0.08 **	44.44	4.98 ± 0.62	15.02

Note: Compared with the model group, * *p* < 0.05, ** *p* < 0.01; comparison with the normal group, ^∆∆^ *p* < 0.01.

**Table 6:** The entropy value method of drug efficacy index value provides a comprehensive score.

Time (h)	Pharmacodynamic Index		Combined Weighted Score
	Lung Index	Virus Volume	S
0.083	0.9382	4.3226	0.0643
0.167	0.9411	4.1338	0.0739
0.25	0.9676	4.1517	0.0470
0.5	0.9290	3.8905	0.0963
1	0.9500	4.0863	0.0656
2	0.8960	4.2529	0.1090
4	0.9422	4.4120	0.0597
6	0.8864	3.7578	0.1441
8	0.9242	4.2827	0.0869
10	0.9335	4.3033	0.0756
12	0.9027	4.7299	0.0848
24	0.8816	4.9773	0.0928

**Table 7:** Analysis results of prototype compounds and comprehensive efficacy (S) of *Scutellaria baicalensis* Georgi.

Compounds	The Correlation Degree with S
Baicalein	0.9078
Apigenin-7-glucuronide	0.8946
Baicalin	0.9478
Oroxylin A 7-*O*-beta-d-glucuronide	0.9041
Wogonoside	0.9263
Norwogonin	0.9012
Wogonin	0.9053
Chrysin	0.8705
Oroxylin A	0.8897

**Table 8:** Reference substance concentration gradient and QC sample concentration.

Components	Nominal Concentration (ng/mL)	Concentrations of QC Samples (ng/mL)		
		Low	Medium	High
Baicalein	1.25–50,000.00	12.5	320.0	20,000.0
Apigenin-7-glucuronide	1.25–50,000.00	12.5	320.0	20,000.0
Baicalin	1.25–50,000.00	12.5	320.0	20,000.0
Oroxylin A 7-*O*-beta-d-glucuronide	1.25–50,000.00	12.5	320.0	20,000.0
Wogonoside	6.25–25,000.00	12.5	160.0	10,000.0
Norwogonin	12.5–50,000.00	25.6	320.0	20,000.0
Wogonin	1.25–50,000.00	12.5	320.0	20,000.0
Chrysin	12.5–50,000.00	25.6	320.0	20,000.0
Oroxylin A	12.5–50,000.00	25.6	320.0	20,000.0

## Data Availability

All data generated or analyzed during this study are included in this article.

## Supplementary Materials

The following supporting information can be downloaded at: https://www.mdpi.com/article/10.3390/molecules28166001/s1. Figure S1: Qualitative identification chromatograms of 9 compounds in mouse plasma; Table S1: Qualitative identification of 9 compounds in mice’s plasma; Table S2: Detection results of drug-containing plasma content at different time points (mean ± SD, *n* = 6).
