# Supplementary material for: Multiplex Quantitative Analysis of 9 Compounds of Scutellaria baicalensis Georgi in the Plasma of Respiratory Syncytial Virus-Infected Mice Based on HPLC-MS/MS and Pharmacodynamic Effect Correlation Analysis
Source: Molecules. 2023 Aug 10;28(16):6001. doi: 10.3390/molecules28166001 (PMC10460054; doi:10.3390/molecules28166001)
Supplement: Supplementary file 1 [file molecules-28-06001-s001.zip › molecules-2517767-supplementary.pdf]

Supplementary Materials:

1. Identification of the components of *Scutellaria baicalensis* Georgi in mice plasma

The TIC maps of mice plasma samples collected by high-performance liquid chromatography-electrostatic field orbitrap high-resolution mass spectrometry (HPLC-Orbitrap Exploris 120 MS) and the quasi-molecular ions and fragmentation ion information obtained. Finally, the fragmentation information of the compounds in the blood samples was compared with the secondary fragment ion information of the components of *Scutellaria baicalensis* Georgi to speculate the composition of *Scutellaria baicalensis* Georgi in respiratory syncytial virus (RSV)-infected mice. The total ion current diagram and fragment information of the compounds in mice plasma ware shown in Figure S1, Table S1.

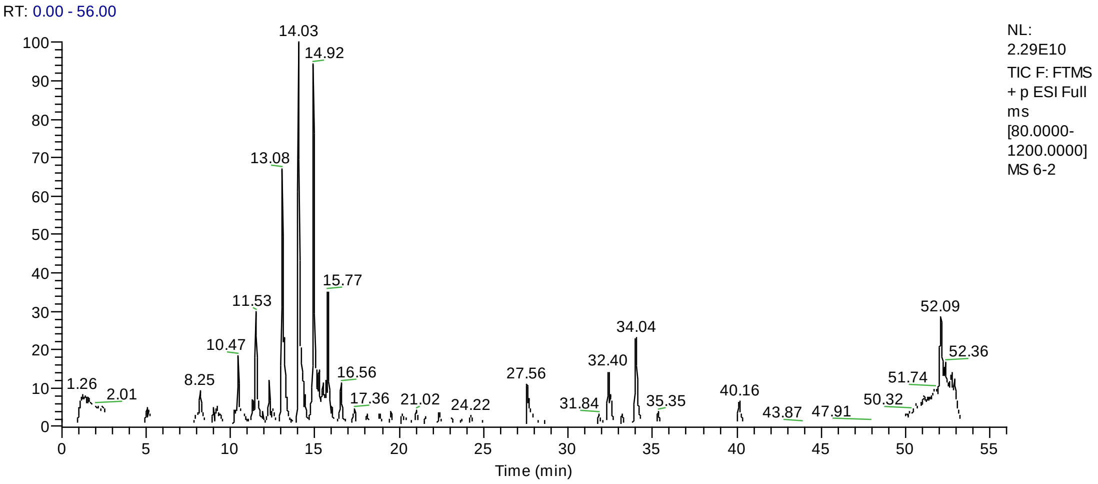

Figure S1. Qualitative identification chromatograms of 9 compounds in mice plasma

Table S1. Qualitative identification of 9 compounds in mice plasma

NO.	Molecular formula	Measured value	Secondary ion	compounds	ESI Mode
1	$C_{15}H_{10}O_5$	271.06	253.04, 123.00	Baicalein	$^{+/-}$
2	$C_{21}H_{18}O_{11}$	447.09	271.05,253.05,169.01,123.00	Apigenin-7-glucuronide	$^{+/-}$
3	$C_{21}H_{18}O_{11}$	447.09	271.05,253.04,169.01,123.00	Baicalin	$^{+/-}$
4	$C_{22}H_{20}O_{11}$	461.10	285.07, 270.05	Oroxylin A 7-O-beta-D-glucuronide	$^{+/-}$
5	$C_{22}H_{20}O_{11}$	461.10	285.07, 270.05	Wogonoside	$^{+/-}$
6	$C_{15}H_{10}O_5$	271.05	253.05	Norwogonin	$^{+/-}$
7	$C_{16}H_{12}O_5$	285.07	270.05, 168.00	Wogonin	$^{+/-}$
8	$C_{15}H_{10}O_4$	255.06	153.07	Chrysin	$^{+}$
9	$C_{16}H_{12}O_5$	285.07	270.05, 168.00	Oroxylin A	$^{+/-}$
10	$C_8H_{13}N_3O_4S$	248.14	121.10，85.02	Tinidazole (IS)	$^{+}$

Table S2. Detection results of drug-containing plasma content at different time points (mean ± SD, *n* = 6).

Time (h)	Baicalein	Apigenin-7-glucuronide	Baicalin	Oroxylin A 7-O-beta-D-glucuronide	Wogonoside	Norwogonin	Wogonin	Chrysin	Oroxylin A
0.083	235.76±21.97	4725.60±214.70	2434.28±151.56	462.93±10.74	553.37±9.22	28.17±2.96	344.59±1.64	0	13.47±9.43
0.167	573.93±23.68	6986.86±153.57	4607.45±318.09	603.78±13.98	1829.58±102.08	94.18±5.68	981.37±13.72	0	745.77±35.54
0.25	437.59±15.17	4610.60±192.08	2031.56±103.57	143.83±6.73	928.49±33.77	23.48±0.05	471.39±1.78	0	63.52±1.32
0.5	243.84±16.32	4344.98±173.73	2696.64±110.33	315.26±12.78	776.20±41.04	13.88±4.73	222.21±6.76	10.37±2.12	40.44±4.81
1	208.98±14.58	1590.11±125.69	2474.37±225.92	138.74±3.62	1078.05±63.01	12.42±7.14	181.04±4.74	15.38±4.94	160.88±15.86
2	25.73±5.59	2934.42±221.23	2620.76±117.82	315.26±12.78	1333.51±38.28	0	10.35±1.79	21.43±4.86	75.84±5.43
4	43.00±8.66	1037.07±106.48	1587.10±169.68	412.32±18.42	1372.78±53.34	14.29±9.94	71.39±17.8	12.37±2.12	486.23±8.65
6	792.15±41.3	9344.98±327.73	8607.45±428.09	1008.71±54.19	3829.58±182.08	113.88±4.73	2664.55±129.18	60.64±2.86	1063.52±101.32
8	184.80±29.24	2958.20±207.61	1856.98±113.03	286.84±8.27	1825.89±177.13	21.94±0.43	97.31±2.71	0	300.68±17.25
10	120.93±15.03	896.04±55.63	569.32±20.22	160.51±5.17	318.50±23.92	16.07±3.80	14.65±8.42	0	19.18±2.56
12	9.28±5.01	47.32±11.82	39.89±7.45	29.40±0.71	188.85±19.38	0	3.41±5.28	0	13.60±7.94
24	1.42±5.86	23.55±2.60	15.44±5.62	0	0	0	0	0	0
